# Optimization of An Enveloped Virus Surrogate, Bacteriophage Phi6, Recovery from Hands

**DOI:** 10.1007/s12560-025-09637-3

**Published:** 2025-03-04

**Authors:** Francis Torko, Kristen E. Gibson

**Affiliations:** Department of Food Science, Center for Food Safety, University of Arkansas System Division of Agriculture, Fayetteville, AR 72704 USA

**Keywords:** SARS-CoV-2, Recovery, Surrogate, Contact transmission, Phi 6

## Abstract

Surfaces contaminated with enveloped viruses, such as severe acute respiratory syndrome coronavirus 2 and influenza virus, can potentially spread illness via hand contact. Often, the efficacy of hand hygiene interventions relies on virus recovery from hands. However, the recovery of bacteriophage phi6 (Φ6), a recommended surrogate for enveloped viruses, from the entire hands using the ASTM E2011-21 standard has not been optimized. For Φ6 recovery from the hands, three eluents [lysogeny broth (LC), tryptic soy broth (TSB), and 1.5% beef extract (BE)] and three recovery methods [glove juice method (GJM), hand rinsing, and modified dish method] were examined. The effects of inoculum application on either the palmar surface or the whole hand were compared, and virus recovery was assessed under wet and dry conditions to identify the optimal combinations for maximizing Φ6 recovery. Statistical differences among methods, inoculum application, and recovery types were identified. While no statistical difference was observed among the eluents (*P* = 0.281), LC demonstrated the highest Φ6 recovery efficiency, while TSB and BE had comparable recoveries. Two-way interaction effects were observed between method type vs. application type (*P* ≤ 0.05), method type vs. recovery type (*P* ≤ 0.05), and application type vs. recovery type (*P* ≤ 0.05), indicating these factors influencing one another. Additionally, no Φ6 recovery was obtained for the dry basis recovery type and the GJM method type. Based on the present study, to maximize Φ6 recovery from the hands during hand hygiene studies, inoculum should be applied to the palmar surface and recovered while it is still wet using LC.

## Introduction

Enveloped viruses, such as Severe Acute Respiratory Syndrome Coronavirus 2 (SARS-CoV-2) and human influenza, pose significant risks to public health due to their ability to transfer via a variety of routes, including direct person-to-person contact or indirect contact via contaminated surfaces. Enveloped viruses are the leading causes of viral global epidemics with a significant public health burden (Zeng et al., 2023). Though aerosols or large respiratory droplets are the primary route of transmission for enveloped respiratory viruses, these viruses can also be transmitted by direct contact between an infected person and a susceptible person (Leung, 2021). Indirect contact transmission is also a potential route of transmission of respiratory viruses.

Although it is less likely, enveloped respiratory viruses can nonetheless be transmitted by indirect contact via contaminated surfaces. For instance, infectious influenza A (H1N1) virus can persist on glass surfaces for 66 days at 4 °C and 7 days at 35 °C (Dublineau et al., 2011). Also, avian influenza (H5N1) virus was found to persist on glass and steel at 4 °C under low relative humidity (27.7–46.3%) (Wood et al., 2010). Similarly, SARS-CoV-2, though primarily known to be transmitted via the respiratory route, has the potential to be transmitted via contaminated surfaces (Baker & Gibson, 2022a; Choi et al., 2021; Van Doremalen et al., 2020). For example, a review by Marquès and Domingo (2021) indicated that SARS-CoV-2 can persist on various surfaces (e.g., plastic and stainless steel) ranging from hours to a few days. Given the ability to persist on surfaces, subsequent transmission via contaminated hands and control via available hand hygiene approaches is important to characterize.

The ability to recover viruses from hands is an important first step in the investigation of the efficacy of hand hygiene implements (e.g., soaps, antiseptics) against viral pathogens. Various methods are available for the recovery of viruses from hands during hand hygiene efficacy studies. For example, the American Society for Testing and Materials (ASTM) recommends either the glove juice method (GJM) or hand rinsing method for the recovery of viruses from the entire hands (ASTM International, 2021), and the dish method for the recovery of viruses from fingerpads (ASTM International, 2017). The ASTM methods are mostly focused on the recovery of non-enveloped viruses, though the methods allow for the substitution of other viruses including enveloped viruses. Studies have previously explored the recovery of enveloped viruses from whole hands and fingerpads using GJM and swabbing methods (Baker & Gibson, 2022b; Casanova & Weaver, 2015). These studies also evaluated different eluents for virus recovery.

Casanova and Weaver (2015) utilized ASTM E1174-13 (ASTM International, 2013) and reported 2.4 and 2.6 log plaque forming unit (PFU) loss of bacteriophage phi6 (Φ6) during recovery with the GJM with tryptic soy broth (TSB) and 1.5% beef extract (BE), respectively, followed by 2.8 logs loss for phosphate buffered saline (PBS). The authors stated that BE can maximize the recovery of enveloped viruses during whole hand sampling using the GJM. However, Casanova and Weaver (2015) exclusively used GJM, focused on virus recovery after wet inoculum, and only considered application of the inoculum to the entire hands, without exploring other application methods such as to the palmar surface which is the primary area for hand contact with surfaces. Additionally, Baker and Gibson (2022b) conducted a study on the recovery of Φ6 from fingerpads using four elution buffers and three recovery methods. While the differences were not significant, the dish method resulted in higher virus recovery rates compared to the modified GJM and swabbing methods. Furthermore, viral transport medium, which contained fetal bovine serum, achieved the highest rates compared to PBS, PBS with 0.01% tween, and 0.1 M glycine with 3% BE. However, the recovery was performed on the fingerpads, which do not accurately mimic real-world hand contamination (Baker & Gibson, 2022a).

In addition, there is limited research on the recovery of the enveloped virus surrogate, bacteriophage phi6 (Φ6), from hands using the ASTM E2011-21 standard protocol for the recovery of viruses from the entire hands (ASTM International, 2021). This protocol primarily recommends the use of non-enveloped viruses, which may not reflect recovery for enveloped viruses. Also, the procedure describes inoculum application on whole hands and virus recovery based on dried inoculum, where the inoculum is allowed to dry before recovery. However, the application of inoculum to the whole hands may potentially result in lower recovery efficiency. In addition, studies have also shown a virucidal effect of drying on Φ6 survival (Baker & Gibson, 2022b; Bangiyev et al., 2021) which could impact the effectiveness of using the ASTM E2011-21 procedures for Φ6 recovery (ASTM International, 2021).

Establishing an optimized method to recover enveloped viruses from hands is critical to effectively undertake hand hygiene efficacy studies for the control of these viruses. However, working directly with highly contagious pathogens poses significant risks to the individuals involved and so surrogate microorganisms are often used to study the fate of such pathogens (Sinclair et al., 2012). Therefore, to investigate the optimal recovery of enveloped viruses from hands, bacteriophage Φ6, a double-stranded RNA virus that belongs to the family *Cystoviridae* (Gottlieb & Alimova, 2023; Mäntynen et al., 2023), was utilized in the present study. More specifically, Φ6 is the recommended surrogate to study enveloped viruses that cannot be easily studied due to safety concerns, especially while using human volunteers (Baker & Gibson, 2022b; Fedorenko et al., 2020; Whitworth et al., 2020; Wood et al., 2020). Various eluents, recovery methods, inoculum application types, and inoculum recovery bases (wet vs. dry) were investigated to maximize the recovery of enveloped viruses from the hands based on the ASTM E2011-21 standard protocol with some modifications (ASTM International, 2021).

## Methods

### Host Cultivation and Virus Propagation

Φ6 was propagated using previously described methods (Baker & Gibson, 2022b). Briefly, Φ6 was grown in *Pseudomonas syringae* pathovar *phaseolicola* (Pph) on lysogeny agar/broth (LC; 10 g/L NaCl, 10 g/L tryptone, 5 g/L yeast extract, pH 7.5). Virus stock was produced via the double agar overlay assay (DAL). For this, a single colony of Pph was cultured overnight in 25 mL of LC broth. Subsequently, 250 μL of Pph overnight culture was mixed with 100 μL of virus stock (~ 10 logs PFU/mL) in 5 mL of LC soft agar, poured onto corresponding LC agar plates and incubated overnight at 25 °C. Plates with a lacy-lawn appearance were harvested, filtered, and stored at 4 °C or − 20 °C for later use.

### Hand Preparation

As previously described (Baker & Gibson, 2022b), hands were prepared by washing hands with non-antimicrobial soap (Equate Hand Soap; Sam’s Club, Fayetteville, Arkansas) for 60 s under running tap water and dried thoroughly with paper towels. Approximately 5 mL of 80% ethanol was then added to the hands, rubbed over the entire hands until dry, and then rinsed with approximately 200 mL of sterile deionized water followed by drying with a paper towel. Three volunteers from within our research group were used for the entirety of the study. The volunteers had no skin irritations to latex or any of the ingredients used for eluent preparations and had intact skin. Volunteers were asked to avoid using antimicrobials before experiments though this cannot be verified.

### Hand Inoculation with Phi6

Hand inoculation was carried out according to ASTM E2011-21, with some modifications (ASTM International, 2021). After hand decontamination, 1 mL of Φ6 suspension, in the presence of ASTM tripartite organic matter (in the ratio 5% bovine serum albumin (BSA), 20% bovine mucin, and 7% yeast extract), at approximately 8 log PFU/mL was inoculated onto the palm of the left hand and rubbed over the entire surface (whole hand) of both hands or the palmar surface of both hands and recovered after 10 s (wet basis recovery) or 90 s and allowed to dry for another 90 s (dry basis recovery) before recovery. One milliliter of inoculum was used as opposed to ASTM E2011-21 recommended 1.5 mL to minimize spillage of inoculum during hand rubbing (ASTM International, 2021). Following inoculation, a total of 70 mL of LC, TSB, or 1.5% BE was used to recover the virus by the GJM, hand rinsing, or modified dish method.

### Virus Recovery by Glove Juice Method

The ASTM E2011-21 was employed for hand recovery with the GJM, with slight modifications (ASTM International, 2021). Each hand was covered with a loose-fitting sterile disposable glove, and 35 mL of eluent (LC, TSB, or 1.5% BE) was added to each glove and securely tightened above the wrist. Hands were massaged for 1 min to recover the virus. The sampling solutions were transferred to a sterile sample container. The samples were assayed to determine the concentration of the recovered virus using DAL. Thirty-five milliliters of eluent was added to each glove instead of 40 mL, as suggested by ASTM E2011-21, to minimize spillage of the eluent containing recovered Φ6 from the gloves.

### Virus Recovery by Hand Rinsing

Φ6 recovery using the hand rinsing procedure as described by ASTM E2011-21 was employed (ASTM International, 2021). After hand inoculation, hands were placed over a sterile funnel (Fisherbrand Large LDPE Funnel, 31.1 cm diameter × 29.21 cm height, Fisher Scientific, Rochester, NY) located above a sterile container (Media/Storage Bottle, 100/150 mL, VWR, Radnor, PA), and 70 mL of eluent was slowly added to the hands within 1 min while the hands were rubbed together to recover the virus. The eluate was assayed to determine the amount of virus recovered using DAL. Seventy milliliters of eluent was used in place of 20 mL, as suggested by ASTM E2011-21, to ensure maximum removal of virus from hands**.**

### Virus Recovery by Dish Method

The dish method recovery was performed according to the ASTM E1838-17 protocol with some modifications for whole hand recovery (ASTM International, 2017). After inoculation of hands with Φ6, hands were pressed onto the bottom of a sterile container containing 70 mL of eluent and rubbed continuously for 1 min. For the whole hand application method, the palmar surface and dorsal side of the hands were pressed and rubbed simultaneously for 1 min. The eluate was then transferred into a sterile container and assayed to quantify virus recovery using DAL.

### Data Analysis

All experimental units were performed in triplicates using different subjects each time. Samples were plated in duplicates and results were averaged to obtain a single value for an experimental unit. PFU/mL values were log_10_ transformed into a log PFU/mL and used for all analyses. Values that were below the detection limit (1 PFU/mL) on two-fold zero dilution plates were assigned 1 PFU/mL and log_10_ transformed to 0 log PFU/mL. The response variable, recovery efficiency, was calculated by dividing the log PFU of the recovered virus by the log PFU of the expected virus to be recovered and then multiplied by 100. A balanced complete block design was used for this study where each treatment occurred in each block the same number of times. Subjects were treated as blocks and the variance accounted for by the different subjects was computed to determine variability among the volunteers. Where *P* ≤ 0.05, we concluded that there is a statistical significance. All data analyses were performed using the R statistical software version 4.2.2. A linear effect model of main effects and two-way interaction effects was built, after which analysis of variance (ANOVA) of the model was performed to detect the statistical significance and potential interaction effect of the factors explored. Arithmetic mean ± standard deviations were calculated to determine the overall average and variability across various combinations of the factor levels.

## Results

**Fig. 1:**
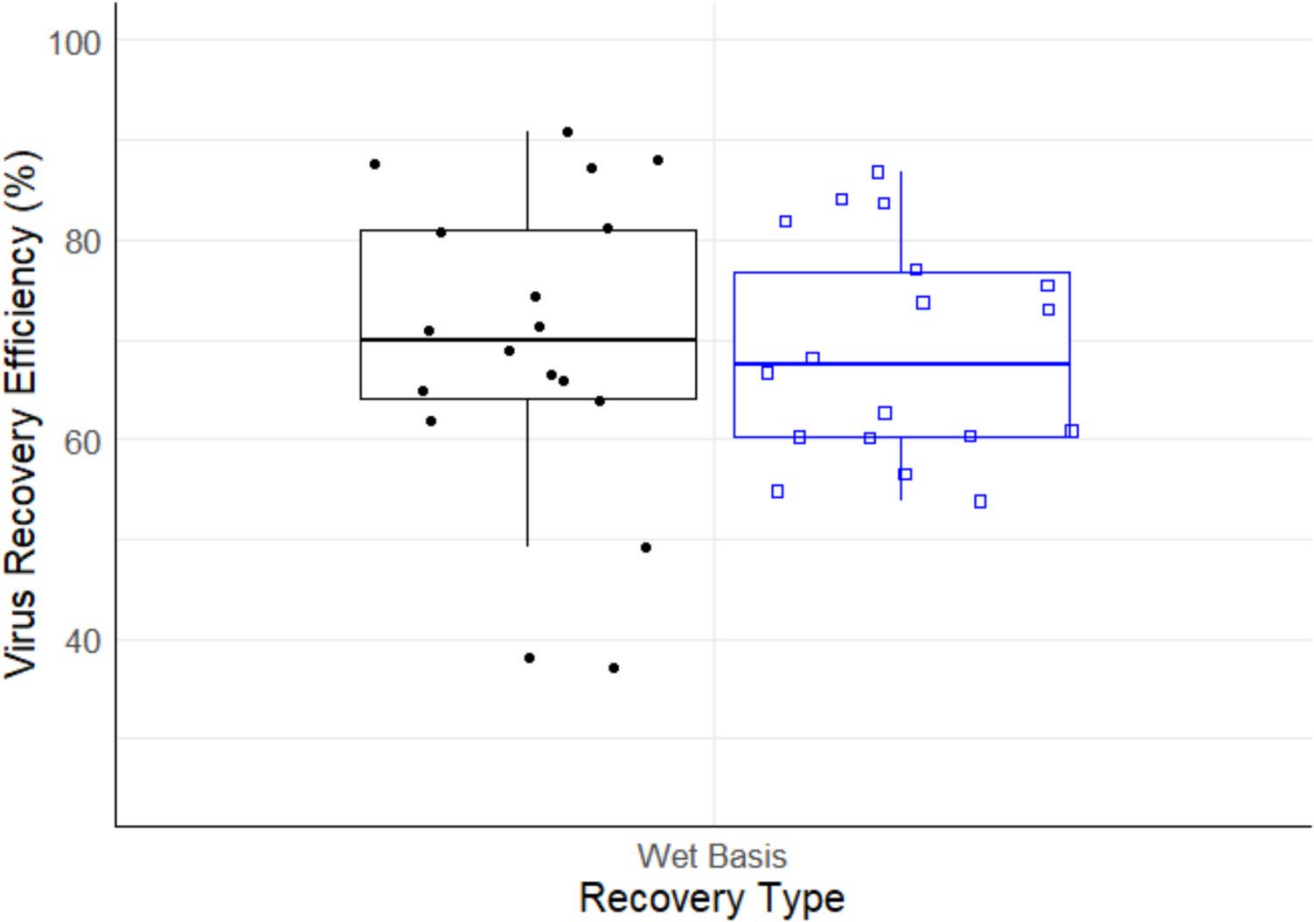
Recovery efficiency of Φ6 based on wet basis recovery type for the dish (black box plot, black dot), glove juice method (red box plot, red open triangle), and rinse (blue box plot, blue open square). *GJM* glove juice method. The boxplot middle line represents the median, the hinges represent 25th and 75th percentiles, and whiskers represent values within 1.5× interquartile range from the 25th and 75th percentiles, respectively (Color figure online)

**Fig. 2:**
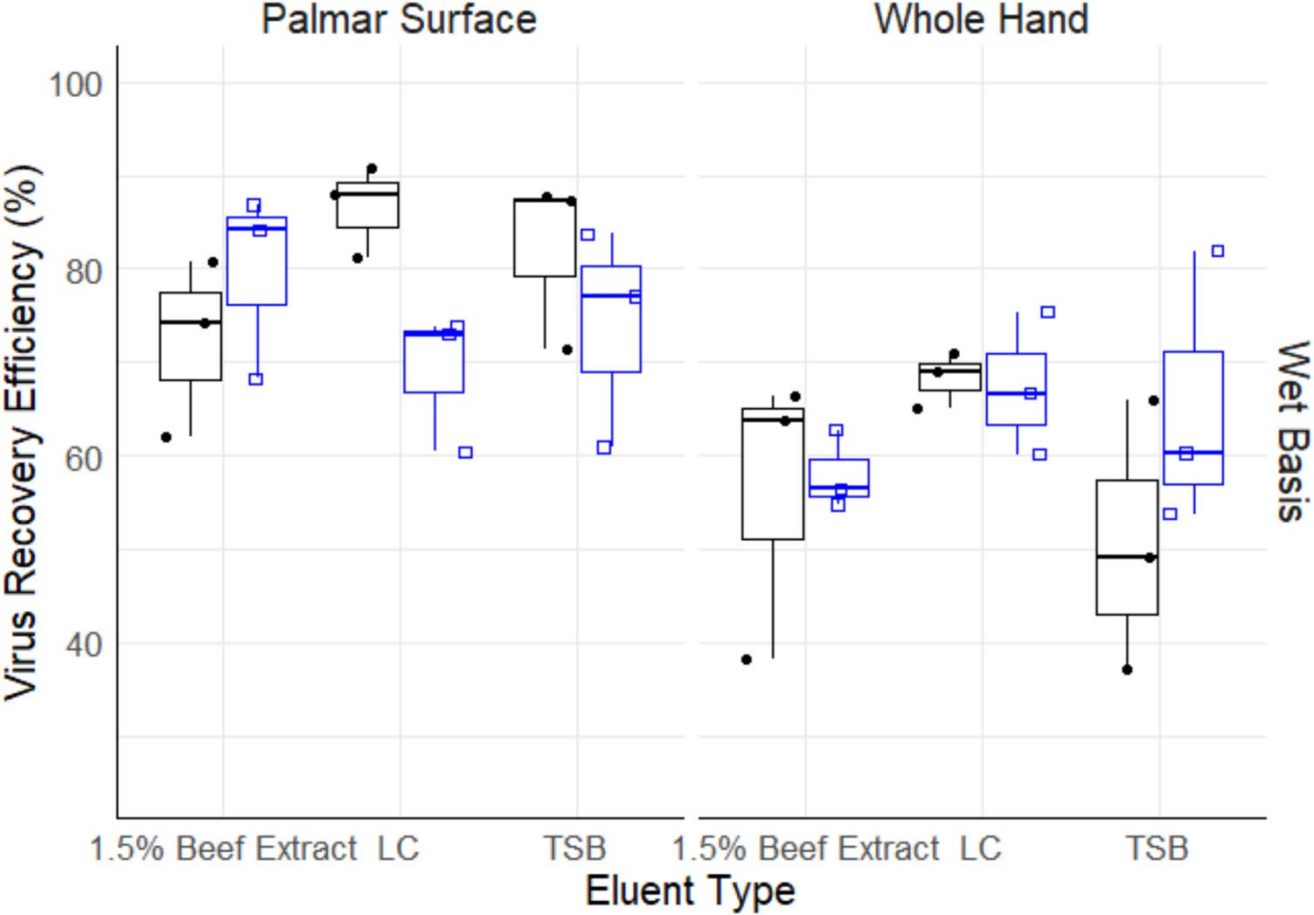
Wet basis recovery efficiency of Φ6 using 1.5% beef extract, LC, and TSB, over the palmar surface and whole hand application types for dish (black box plot, black dot), GJM (red box plot, red open triangle), and rinse (blue box plot, blue open square). *GJM* glove juice method. The boxplot represents the minimum (whisker below), median (middle line), and maximum values (whisker above) (Color figure online)

The overall amount of virus suspended, expected to be recovered, and the actual amount recovered from volunteers’ hands was 8.05 ± 0.18, 6.20 ± 0.18, and 1.43 log PFU/mL, respectively. Treating volunteers as a block, ANOVA results indicated no significant difference in recovery efficiency across volunteers. Further analysis performed by estimating the variance components for volunteers indicated that there was minimal variation in recovery efficiencies. ANOVA output for main effects indicated statistical significance for method type (*P* ≤ 0.05), application type (*P* ≤ 0.05), and recovery type (*P* ≤ 0.05). Two-way interaction effects indicated significant interaction effects between method type vs. application type (*P* ≤ 0.05), method type vs. recovery basis (*P* ≤ 0.05), and application type vs. recovery basis (*P* ≤ 0.05). This suggests that the mean recovery efficiency of method types (dish, GJM, rinse) depends on the type of inoculum application (palmar surface vs whole) as well as the recovery basis (wet vs dry basis) performed on hands. Moreover, the mean recovery efficiency of the inoculum application type depends on whether a wet basis or dry basis recovery was performed. Table 1 presents the average recovery efficiencies (%) for all levels of recovery basis, method, inoculum application, and eluent types. Figure 1 graphically displays the wet basis recovery efficiencies (%) by method type, while Fig. 2 illustrates the efficiencies for different eluent types across inoculum application types.

**Table 1:** Average Φ6 recovery efficiencies (%) based on method, application, recovery, and eluent types

Method	Inoculum application	Recovery basis	Eluent type		
			1.5% BE	LC	TSB
Dish	Palmar surface	Dry	–	–	–
		Wet	72.29 ± 9.54	86.62 ± 4.95	82.07 ± 9.34
	Whole hand	Dry	–	–	–
		Wet	56.16 ± 15.6	68.26 ± 3.02	50.70 ± 14.50
GJM	Palmar surface	Dry	–	–	–
		Wet	–	–	–
	Whole hand	Dry	–	–	–
		Wet	–	–	–
Rinse	Palmar surface	Dry	–	2.77 ± 4.80	–
		Wet	79.73 ± 10.06	69.05 ± 7.50	73.89 ± 11.75
	Whole hand	Dry	–	–	–
		Wet	58.01 ± 4.17	67.40 ± 7.66	65.32 ± 14.74

*GJM* glove juice method, *BE* bovine extract, *LC* lysogeny broth, *TSB* tryptic soy broth

“−” indicates that there were no viruses recovered using this method

### Recovery Efficiency by Recovery Basis

A statistical significance was observed between wet basis and dry basis recoveries. This is because Φ6 was primarily recovered when the inoculum remained wet as opposed to dry. The overall recovery efficiency for wet and dry basis was 46.0 ± 35.0 and 0.15 ± 1.13%, respectively. The large variation observed for wet basis was due to the inability to recover Φ6 for the GJM method type.

### Recovery Efficiency by Eluent Type

Due to the negligible recovery for the dry basis as stated earlier, recovery efficiency for the different eluents was estimated for only wet basis recovery. There was no statistical significance between the eluent types, however, the highest overall recovery efficiency was recorded for LC, followed by TSB and BE. The recovery efficiency for LC, TSB, and BE was 48.6 ± 36.2, 45.3 ± 35.5, and 44.4 ± 34.1%, respectively. Similarly, the high variations observed among eluents were due to the inability to recover virus using the GJM method type. Comparing the eluents for wet basis recovery without the influence of GJM, the recovery efficiencies were 72.8 ± 9.82, 68.0 ± 16.3, and 66.5 ± 13.7%, for LC, TSB, and BE, respectively.

### Recovery Efficiency by Method Type

Due to the insignificant recovery of Φ6 with the dry basis recovery method and the recovery falling below the detection limit for GJM, further analyses based on method type were performed for dish and rinse methods for wet basis recovery. There was no statistical significance between the dish and rinse methods for wet basis recovery (*P* = 0.985). However, after computing marginal means, with p-value adjustment using Tukey correction to control for family-wise error rate, the dish method recovered slightly more virus than the rinse method with 69.3 ± 15.9 and 68.9 ± 10.9% recovery efficiencies, respectively, across all levels of eluent and inoculum application types.

### Recovery Efficiency by Inoculum Application Type

In this study, the inoculum was applied to either the palmar surface or the whole hands prior to recovery. There was a significant difference in recovery efficiency between the palmar surface and the whole hand applications. As indicated earlier, the recovery efficiency of application type is also dependent on the method used to recover viruses from the hands. For wet basis recovery and palmar surface application, the dish method and rinse method types achieved recovery efficiencies of 80.3 ± 9.53 and 74.2 ± 9.76%, respectively. Similarly, these methods showed recovery efficiencies of 58.4 ± 13.3 and 63.6 ± 9.57%, respectively, for whole hand application and wet basis recovery. Recovery efficiency was found to be statistically different among the palmar surface and whole hand application types, with the former recording higher recovery efficiency. Without considering GJM, the palmar surface and whole hand recovery efficiencies were 77.3 ± 9.87 and 61.0 ± 11.5%, respectively. Overall, for both the palmar surface and whole hand inoculum application types, LC, dish, and wet basis had the highest recovery efficiency for eluent, method, and recovery types (Table 1).

## Discussion

The present study emphasizes that the recovery of enveloped viruses, using Φ6 bacteriophage as a surrogate, is significantly affected by a range of factors. Previous studies have demonstrated how factors such as eluent, recovery method, humidity, temperature, and inoculum droplet size impact the recovery of enveloped viruses from human skin (Baker & Gibson, 2022b; Casanova & Weaver, 2015; Prussin et al., 2018; Thomas et al., 2014). The ability to optimize Φ6 recovery from the hands is crucial for establishing transfer rates and virus persistence as well as performing hand hygiene efficacy studies.

For optimization of Φ6 recovery from the hands, wet and dry-based recovery was evaluated. For a wet basis, the inoculum was applied to the hands and recovered after 10 s while the inoculum was still wet on the hands. The dry basis recovery was performed by application of inoculum on the hands, rubbing for 90 s, and allowing it to dry for another 90 s before virus recovery, as recommended by ASTM E2011-21 (ASTM International, 2021). Previous studies have demonstrated a detrimental effect of drying on virus survival (Baker & Gibson, 2022b; Bangiyev et al., 2021; Firquet et al., 2015). For example, Grayson et al. (2009) reported an immediate reduction of 3 to 4 log reduction of influenza A virus (an enveloped virus) after 2 min of inoculum drying on hands as compared to a baseline of 7 log PFU/mL. Consequently, 6 out of 20 participants did not have a culture-detectable infective virus across experimental replications. The reduction in influenza A virus due to drying is similar to Baker and Gibson (2022b), where even though inoculum remained wet for 15–20 min after inoculation, a log PFU loss of 4.57 ± 0.87 and 5.58 ± 0.71 was observed after 15 and 30 min (including 5 min of wait time before inoculation), respectively.

Similarly, a significant impact of drying on Φ6 survival, suspended in water microdroplets, was observed when more than a 4 log reduction in viability was reported by Fedorenko et al. (2020) due to drying after 14 h of incubation under different relative humidity levels. Sun et al. (2020) also investigated the stability of SARS-CoV-2 under different conditions, including wet and dry, and the authors concluded that, while the virus can survive under wet and dry conditions, the dry environment may be less favorable for its survival. Results obtained from this study align with the virucidal effect that drying has on Φ6 infectivity. For the dry basis recovery, there was a mean loss of 6.17 log PFU as compared to a baseline of 6.18 log PFU, affirming the significant virucidal effect that drying has on virus infectivity. The high log loss of virus may be a result of the combined effect of rubbing and drying as recommended by the ASTM E2011-21 standard method (ASTM International, 2021). However, for the wet basis recovery, recovery of Φ6 from hands was achieved.

There was a significant difference in the recovery efficiency across methods. The dish method exhibited higher recovery efficiency over the rinse methods, across various eluents and application types. This observation is similar to the results by Baker and Gibson (2022b) for the recovery of Φ6 from inoculated fingerpads at different time points. The authors established that, though there was no statistical difference among the methods explored (dish, modified GJM, and vigorous swabbing), the dish method had higher recovery over the modified GJM at recovery time points of 0 and 30 min. The authors also mentioned their preference for the dish method over the modified GJM and vigorous swabbing methods due to ease of use and collection of eluates after the recovery step.

Previous studies explored the use of the GJM for the recovery of Φ6 from hands (Casanova & Weaver, 2015; Wolfe et al., 2017). Following the ASTM E1174-13, Casanova and Weaver (2015) applied 1.5 mL of inoculum to the subjects’ hands, rubbed for 20 s, and repeated the process two more times to obtain a total of 4.5 mL inoculum, followed by immediate recovery. Meanwhile, Wolfe et al. (2017) performed Φ6 recovery using a modified GJM where volunteers placed their hands in a WhirlPak bag containing 75 mL of eluent and were asked to rub their own hands for 30 s followed by a researcher massaging the bag and the hand together for another 30 s. Similarly, Baker and Gibson (2022b) also utilized a modified GJM approach to recover Φ6 from the fingerpads. While recovery was observed in this study, the method was modified by using a 2 × 3″ poly bag for the recovery. The differences in inoculum application and recovery procedure, including the use of different sampling materials, may influence the ability to recover Φ6 from the hands using the GJM.

Different eluents were also explored for optimization of Φ6 recovery from the hands. Previous studies have demonstrated the potential of eluent composition to maximize virus recovery (Carducci et al., 2002; Casanova & Weaver, 2015). For instance, Casanova and Weaver (2015) compared BE, 0.01% Tween 80 in PBS, and 9% NaCl for recovery of Φ6 from the whole hands. It was established that BE had the lowest virus loss (2.8 log reduction), followed by 0.01% Tween 80 in PBS (2.9 log reduction) and 9% NaCl (3.8 log reduction). Similarly, Carducci et al. (2002) investigated the recovery of hepatitis C virus on surfaces in hospital settings using two eluents: 3% BE at pH 9 and 1% BSA with 0.85% NaCl. Results indicated that 3% BE had greater efficiency with 76% recovery when compared to 1% BSA with 0.85% NaCl which had only 13% recovery.

In the present study, although there were no statistically significant differences among the eluents, the highest recovery efficiency was observed using LC, followed by TSB and BE. These findings align with the results from Casanova and Weaver (2015), who also compared TSB, BE, and PBS. It was found that TSB had the lowest virus loss, followed by BE and then PBS with 2.4, 2.6, and 2.8 log reduction, respectively. It is important to note also that, while BE yielded average recovery efficiencies lower than TSB, the differences were minimal, indicating that recoveries among these eluents are comparable in the present study. Specifically, TSB performed better than BE under certain conditions (dish, palmar surface, and wet basis; and rinse, whole hand, wet basis), while BE outperformed TSB in other scenarios as seen in Table 1. These observations suggest that the optimal choice of eluent, whether BE or TSB, for the recovery of Φ6 may vary depending on the specific conditions and desired efficiency of the recovery process.

For the wet basis, the palmar surface inoculation resulted in a higher recovery of Φ6 compared to whole hand inoculation. This finding suggests that palmar surface inoculation would be the preferred application type to maximize Φ6 recovery as compared to the whole hand which is recommended by ASTM E2011-21 (ASTM International, 2021). Also, the palmar surface is the primary area of the hand that is in contact with surfaces, with the potential to result in transmission of viral pathogens making it an important application type to consider in hand hygiene studies. Overall, the dish method, LC, and wet basis recovery had the highest recovery efficiencies for palmar surface and whole hands application types in the present study.

Several challenges were identified in the recovery of Φ6, including the formation of foam particles. During the mixing of samples to achieve homogeneity before dilution, foam particles formed, potentially entrapping virus particles and diminishing recovery efficiencies. Additionally, excessive foam formation occurred during the GJM recovery process due to hand massaging. The formation of foam could also be attributed to the protein-rich content of the eluents used. Another significant challenge is recovering all eluate volumes from the glove post-sampling. These factors could adversely affect the overall recovery of virus from hands.

The present study underscores that multiple factors influence the recovery efficiencies of enveloped viruses from human hands. It is essential to establish an optimal eluent and method of recovery and to determine the appropriate recovery basis and inoculum application type for enveloped virus recovery. Based on this study, to effectively recover enveloped viruses from hands, the inoculum should be applied to the palmar surface and recovered while still wet using LC as an eluent. Allowing the inoculum to dry before recovery was observed to adversely affect the survivability of Φ6, and rubbing may further exacerbate the inactivation of the virus.

## Data Availability

No datasets were generated or analyzed during the current study.
